# Selective Reduction of Ca^2+^ Entry Through the Human NMDA Receptor: a Quantitative Study by Simultaneous Ca^2+^ and Na^+^ Imaging

**DOI:** 10.1007/s12035-024-03944-9

**Published:** 2024-01-19

**Authors:** Tiziano D’Andrea, Maria Cristina Benedetti, Lucia Monaco, Alessandro Rosa, Sergio Fucile

**Affiliations:** 1 Department of Physiology and Pharmacology, Sapienza University of Rome, Rome, Italy; 2 Department of Biology and Biotechnologies “Charles Darwin”, Sapienza University of Rome, Rome, Italy; 3 Center for Life Nano- & Neuro-Science, Fondazione Istituto Italiano di Tecnologia (IIT), Rome, Italy; 4 IRCCS Neuromed, Pozzilli, IS Italy

**Keywords:** Excitotoxicity, Neurodegeneration, Neuroprotection, Ca^2+^ permeability, Negative allosteric modulator, Human iPSCs

## Abstract

Excessive Ca^2+^ influx through N-methyl-D-aspartate type glutamate receptors (NMDAR) is associated with excitotoxicity and neuronal death, but the inhibition of this receptor-channel causes severe adverse effects. Thus, a selective reduction of NMDA-mediated Ca^2+^ entry, leaving unaltered the Na^+^ current, could represent a valid neuroprotective strategy. We developed a new two-fluorophore approach to efficiently assess the Ca^2+^ permeability of ligand-gated ion channels, including NMDARs, in different conditions. This technique was able to discriminate differential Ca^2+^/Na^+^ permeation ratio through different receptor channels, and through the same channel in different conditions. With this method, we confirmed that EU1794-4, a negative allosteric modulator of NMDARs, decreased their Ca^2+^ permeability. Furthermore, we measured for the first time the fractional Ca^2+^ current (P_f_, i.e. the percentage of the total current carried by Ca^2+^ ions) of human NMDARs in the presence of EU1794-4, exhibiting a 40% reduction in comparison to control conditions. EU1794-4 was also able to reduce NMDA-mediated Ca^2+^ entry in human neurons derived from induced pluripotent stem cells. This last effect was stronger in the absence of extracellular Mg^2+^, but still significant in its presence, supporting the hypothesis to use NMDA-selective allosteric modulators to lower Ca^2+^ influx in human neurons, to prevent Ca^2+^-dependent excitotoxicity and consequent neurodegeneration.

**Supplementary Information:**

The online version contains supplementary material available at 10.1007/s12035-024-03944-9.

## Introduction

The N-methyl-D-aspartate type glutamate receptors (NMDAR) are Ca^2+^-permeable glutamate-gated cationic channels expressed throughout the CNS, where they play key physiological roles in several processes, such as synaptic function and plasticity, and therefore learning and memory [1]. The flow of current through NMDAR needs two coincident events: the presence of the agonist molecules (glutamate and the coagonist, glycine or d-serine), and the membrane depolarization, removing the channel block caused by Mg^2+^ [2]. Activated NMDAR induces a slow synaptic current that allows a substantial influx of external Ca^2+^ [1, 3]. The high Ca^2+^ permeability of NMDAR is fundamental in several processes, leading to multiple short-term or long-term changes in the synaptic strength of the postsynaptic neuron [1]. However, an imbalance in glutamate homeostasis and the consequent overactivation of glutamate receptors, in particular in conditions of energy availability reduction and increased oxidative stress, can damage and kill neurons [4]. This excitotoxic mechanism is mainly the response to a massive influx of Ca^2+^ through NMDAR [2] and voltage-dependent Ca^2+^ channels that cause reactive oxygen species (ROS) release and consequent mitochondrial dysfunction [4]. Classic examples of excitotoxic neuronal degeneration in humans include severe epileptic seizures and stroke, but also neurodegenerative processes in chronic neurodegenerative diseases, including Alzheimer’s disease, Parkinson’s disease, Huntington’s disease, and amyotrophic lateral sclerosis (ALS) [4]. Based on the pathogenic concept of excitotoxicity, NMDAR has been a longstanding therapeutic target for rational drug design that could be used against these otherwise heterogeneous and complex pathologies [5]. For instance, one of the few approved drugs against Alzheimer’s disease is memantine, a partial open-channel blocker of NMDAR. However, eliminating the NMDAR current is not compatible with life, and even a partial block is sufficient to generate several adverse effects [6]. A suitable but still unexplored therapeutic strategy could be the selective reduction of Ca^2+^ influx through NMDAR, leaving unaltered the Na^+^ current. During the last three decades, the Ca^2+^ permeability of ligand-gated ion channels has been quantified in terms of fractional Ca^2+^ current (P_f_, the percentage, carried by Ca^2+^ ions, of the total current flowing through an ion channel) with an experimental approach combining simultaneously both electrophysiological and Ca^2+^ imaging techniques [7]. This technique has been used to measure the P_f_ of several cationic ion channels, such as nicotinic acetylcholine receptors (nAChRs; [8]) and ionotropic glutamate receptors [9]. The NMDAR’s P_f_ (~ 12%) is the highest among all known glutamate receptors [9]. The same technique was used in our laboratory to demonstrate that the Ca^2+^ permeability of human muscle nAChRs could be pharmacologically decreased and to show that the therapeutic effect of several molecules used to treat slow-channel myasthenic syndromes was due to their ability to decrease selectively the excess of Ca^2+^ entry at the neuromuscular junction [10].

Furthermore, we demonstrated that extracellular mild acidosis decreases the P_f_ of human NMDARs, reducing the relative Ca^2+^/Na^+^ influx ratio through NMDARs [11]. This evidence pushed us to identify a suitable molecule able to reduce Ca^2+^ influx through NMDAR without affecting Na^+^ influx, to prevent or decrease the excitotoxic Ca^2+^-dependent neuronal damage. Strong support in this direction has been made by Traynelis group, which designed several positive and negative allosteric modulators for the human NMDARs using bioinformatical resources [12]. One of those molecules, the EU1794-4, is a negative allosteric modulator able to reduce single-channel conductance and Ca^2+^ permeability of the human NMDAR formed by the GluN1/GluN2A subunits [13]. In the present study, we aim to demonstrate the possibility of reducing pharmacologically the Ca^2+^ permeability of human NMDAR and reduce NMDAR-mediated Ca^2+^ entry in human iPSC-derived neurons, with an innovative approach based on simultaneous Ca^2+^ and Na^+^ fluorescence imaging [14], providing a faster and more efficient method to determine alterations of Ca^2+^ flow through ligand-gated ion channels.

## Materials and Methods

### Cell Culture and Transfection

HeLa cells and Hek293T cells were grown in Dulbecco’s Modified Eagle Medium (DMEM) supplemented with 10% heat-inactivated FBS and 1% penicillin–streptomycin, at 37 °C in a 5% CO_2_ humidified atmosphere. Cells were plated on cover slides (1 × 10^5^ cells/ml) and transiently transfected 24 h later using Lipofectamine 3000 (Thermofisher, Life Technologies Italia, Italy) according to the manufacturer’s protocol, adding 0.5 μg human NR1 and NR2A cDNA subtype per well, or 0.5 μg human α_3_β_4_ nAChR cDNA subtype per well. Recordings were carried out 24–36 h following transfection.

### Simultaneous Ca^2+^and Na^+^ Imaging

Measures of [Ca^2+^]_i_ and [Na^+^]_i_ were obtained by time-resolved digital fluorescence microscopy using Ca^2+^ indicator Fura-2 acetoxymethyl ester (Fura-2 AM, Molecular Probes, Life Technologies; excitation 340 and 380 nm, emission 510 nm) and Na^+^ indicator ING-2 acetoxymethyl ester AM (ING-2 AM, Interchim; excitation 488, emission 525). Fura-2 is a ratiometric probe, thus the [Ca^2+^]_i_ variation is quantified in terms of the ratio (R) of the emission obtained from the two excitation wavelengths (F_340/_F_380_ = R). In the present study, the amplitude of agonist-induced Ca^2+^ transients ΔR = (R peak-R basal) is indicated as F_Ca_. [Na^+^]_i_ variation is quantified, using a single excitation wavelength, as ΔF/F_0_, and the amplitude of Na^+^ transient is reported as F_Na_. Cells with Na^+^ transients exhibiting F_Na_ < 0.015 or F_Na_ > 0.05 were discarded. The ratio F_Ca_/F_Na_ has been used to quantify variations of Ca^2+^ selectivity. Hek 293 T cells in culture were incubated with 1 μM Fura-2 AM and 5 μM ING-2 AM for 1 h at 37 °C in culture medium. Then cells were washed and placed in normal external solution (NES) for fluorescence microscopy experiments. Ca^2+^ and Na^+^ transients were elicited in Hek293T cells transfected with NMDAR or in iPSC-derived motoneurons by applying NMDA 200 μM and glycine 50 μM, or ACh in Hek293T cells transfected with human α3β4 nAChR, for 3 s. Ca^2+^ and Na^+^ transients were recorded by acquiring time-resolved sequences (1 Hz) of fluorescence images alternating excitation wavelengths selective for Ca^2+^ (340 nm, 380 nm) and Na^+^ (488 nm). Each image was acquired with an exposition lasting 100 ms and a switch time between 90 and 100 ms. Ca^2+^ and Na^+^ signals were measured for each cell using ROI following cell morphology. Background subtraction was not necessary. In some experiments, before applying agonists, cells were pretreated for 3 s with NES at pH 6.5 or with the negative allosteric modulator EU1794-4, as indicated. During recording, cells were continuously superfused using a gravity-driven perfusion system consisting of independent tubes for control and agonist solutions. NES contained (in mM): 140 NaCl, 2.8 KCl, 2 CaCl_2_, 2 MgCl_2_, 10 glucose, and 10 HEPES; pH value adjusted with NaOH 1 M at 7.3, or 6.5, as necessary. In experiments in 0 Mg^2+^ the composition of the solution was the same, without MgCl_2_. Perfusion tubes were connected to a fast exchanger system (RSC-160; Bio-logic, Claix, France). All experiments were performed using a Quantem 512SC camera, Leica microscope with HCX Apo L40X/0.80 WU-V-I objectives, Omega optical filter cubes (dichroic mirror DMR-07, emission filter 520 ± 50), a rotating prism-based monochromator (Deltaram X). Data sampling and analysis were performed using Metafluor imaging software (Molecular Devices, Sunnyvale, CA, USA).

### Fractional Ca^2+^ Current

The procedure used for P_f_ measurements follows the method proposed by Zhou and Neher in 1993 [8], which has the advantage of being independent of any assumption on ion-permeation properties [9]. All measurements were performed in transfected HeLa cells, which are not electrically coupled by gap junctions. Cells were loaded with cell-impermeant Fura-2 through the patch pipette used to measure NMDA-evoked currents. Recordings of fluorescence signals and whole-cell membrane currents were synchronized, and images were acquired and analysed offline. All optical parameters and digital camera settings were maintained throughout this study to avoid nonhomogeneous data. The changes of [Ca^2+^]_I_ were expressed as ΔF/F (i.e. basal fluorescence), using only one excitation wavelength, 380 nm, to increase the temporal resolution. Determinations were carried out after the basal fluorescence had reached a stable value. Cells displaying a low-basal F380 value were discarded. In order to evaluate P_f_ the F/Q ratio between the fluorescence increase (F) and total charge that had entered the cell at each fluorescence acquisition time (Q) was defined as F/Q = (ΔF/F)/ Q. For each cell, we used the F/Q points corresponding to early times after NMDA application and following a straight line, indicating a direct proportionality between F and Q, and consequently that the Ca^2+^-buffering capability of Fura-2 was not saturated. The F/Q ratio value was then measured as the slope of the linear regression best fitting the F-Q plot. Finally, P_f_ was determined by normalizing the ratio obtained in standard medium (F/Q) to the calibration ratio, measured when Ca^2+^ ions were the only permeant species (F/Q_Ca_): P_f_ = (F/Q) / (F/Q_Ca_). Calibrations were performed on different days throughout the whole experimental period, using NMDA-evoked Ca^2+^ currents. NMDA was applied to each cell only once, to avoid possible variations of conditions upon repetitive applications, such as basal [Ca^2+^]_i_ increase.

### Human iPSCs Culture and Differentiation

The human T12.9 WT-15 iPSC line used in this study has been previously generated and characterized (a kind gift of J. Sterneckert [15]. iPSCs were maintained in Nutristem-XF medium (Biological Industries), with 0.1X Penicillin/Streptomycin (Merck Life Sciences, Nutristem P/S), in Matrigel-coated dishes (hESC-qualified Matrigel; Corning) and passaged every 4–5 days with 1 mg/ml Dispase (Gibco). iPSCs were co-transfected with 4.5 μg of the epB-NIL transposable vector [16] and 0.5 μg of the piggyBac transposase with the Neon Transfection System (Life Technologies), using 100 μl tips in R buffer and the settings: 1200 V, 30 ms, 1 pulse. Selection was carried out for 10 days with 5 μg/ml blasticidin in Nutristem-XF medium, giving rise to a stable cell line (hereafter NIL iPSCs). Differentiation to a neuronal population enriched for spinal motoneurons was induced as previously described in De Santis et al., 2018 and Garone et al., 2019 [16, 17]. Briefly, NIL iPSCs were dissociated with Accutase (Thermo Fisher Scientific) to single cells and replated in Nutristem 0.1 × P/S supplemented with 10 µM Y-27632 ROCK inhibitor (Enzo Life Sciences). The next day differentiation was induced in DMEM/F12 (Dulbecco’s Modified Eagle’s Medium/ Nutrient Mixture F-12 Ham; Merck Life Sciences), 1X Glutamax (Thermo Fisher Scientific), 1X NEAA (Thermo Fisher Scientific), 0.5X P/S and doxycycline 1 µg/ml (Thermo Fisher Scientific) for 2 days. On the third day, the medium was replaced by Neurobasal/B27 (Neurobasal medium, Thermo Fisher Scientific; 1X B27, Thermo Fisher Scientific; 1X Glutamax; 1X NEAA; 0.5X P/S) supplemented with 5 µM DAPT, 4 µM SU5402 (both from Merck Life Sciences) and 1 µg/ml doxycycline for 3 days. At day 5, neuronal progenitors were dissociated with Accutase and plated at a density of 400 × 10^5^ cells onto Matrigel-coated 35 mm dishes for electrophysiological recordings. Cells were plated in Neurobasal/B27 supplemented with 10 µM Y-27632 ROCK inhibitor for the first 24 h, and then maintained in Neurobasal/B27 supplemented with 20 ng/ml L-ascorbic acid (Merck Life Sciences), 20 ng/ml BDNF (PreproTech) and 10 ng/ml GDNF (PreproTech). Cells were used after a differentiation period of 21 days, when they displayed complete action potentials and neurotransmitter-induced whole-cell currents.

### Electrophysiological Recordings

For P_f_ measurements in Hek293T cells, whole-cell currents were recorded at room temperature using borosilicate glass patch pipettes having a tip resistance of 3–5 MΩ filled with the following solution: 140 mM CsCl, 10 mM HEPES and 0.5 mM Fura-2 pentapotassium salt (pH 7.3); measurements were performed at holding potential of -70 mV. Cell capacitance was routinely compensated using the amplifier function and value used to estimate the cell surface. For measurements in neurons, borosilicate glass patch pipettes having a tip resistance of 3–5 MΩ were filled with the following solution: 140 mM KCl, 2 mM MgATP, 5 mM BAPTA, 10 mM HEPES. Whole-cell currents induced by different neurotransmitters were recorded in the voltage-clamp configuration, while evoked action potentials in the current-clamp configuration. During recording, cells were continuously superfused with the same NES described for imaging experiments. Membrane currents and potentials were filtered at 3 kHz upon the acquisition with the HEKA EPC 800 amplifier (HEKA Elektronik, Germany) and analyzed offline.

### Statistics

All data were expressed as mean ± S.D. and analysed using one-way ANOVA or paired t-test, as appropriate. When necessary, the non-parametric Dunn’s one-way ANOVA on ranks was used. In case of significance, all pairwise multiple comparison procedure was used (Holm-Sidak, or Dunn’s method for non-parametric tests). The minimum power of statistical tests was set at 0.8. The significance for all tests was set at p < 0.05.

## Results

### Simultaneous Ca^2+^and Na^+^ Imaging is Able to Discriminate Between Different Ca^2+^ Permeabilities

**Fig. 1:**
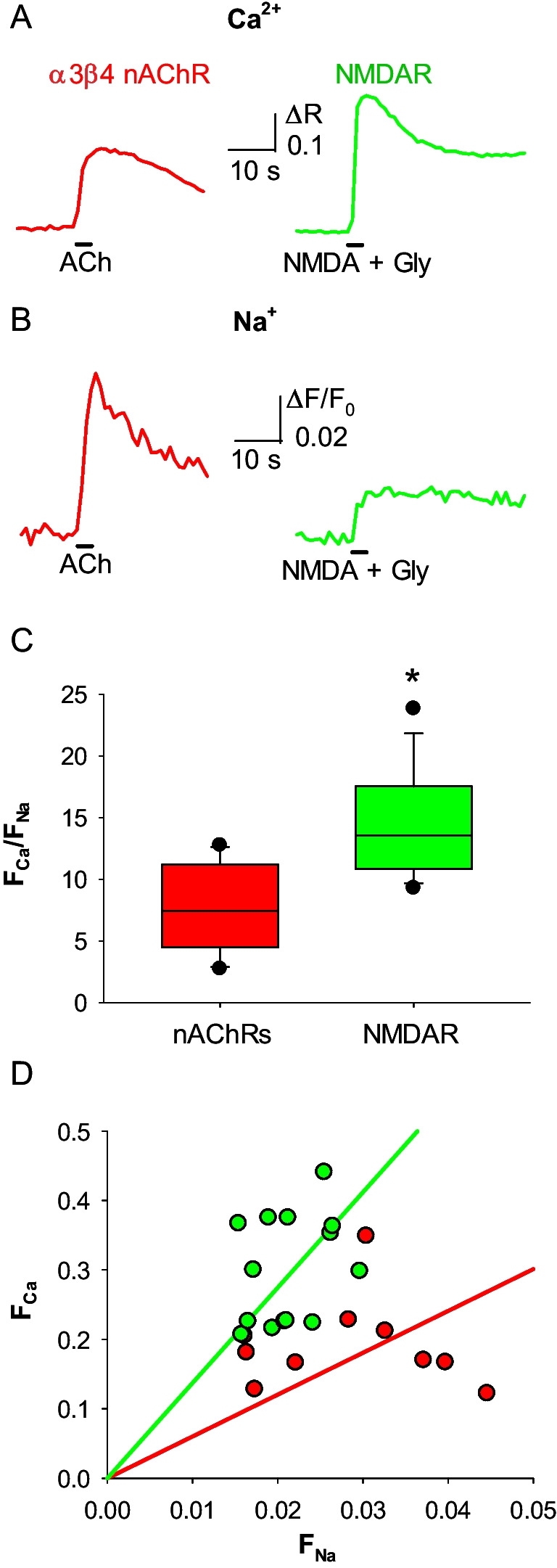
Simultaneous Ca^2+^ and Na^+^ imaging discriminates between ligand-gated ion channels with different Ca^2+^ permeability. **A** representative Ca^2+^ transients recorded in Hek293T cells expressing human α3β4 nAChR (red) and NMDAR (green), elicited respectively by ACh, and NMDA plus glycine. **B** representative Na^+^ transients recorded in Hek293T cells expressing human α3β4 nAChR (red) and NMDAR (green), same cells as A. **C** F_Ca_/F_Na_ for NMDAR (green, *n* = 14) and α3β4 nAChR (red; *n* = 16; *p < 0.001, one-way ANOVA). **D** linear relationships of the distributions of F_Ca_ and F_Na_ values, for NMDAR (green) and α3β4 nAChR (red). Same cells as C

**Fig. 2:**
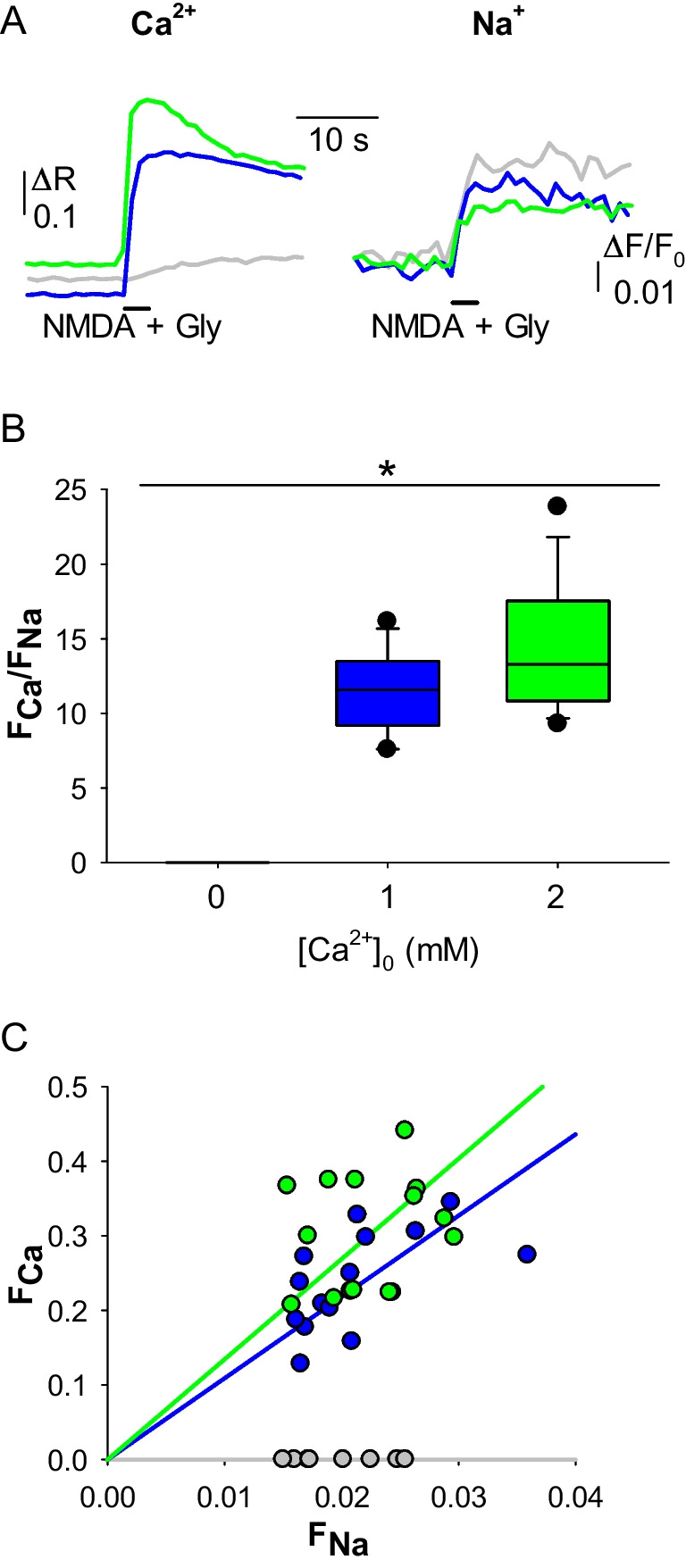
Simultaneous Ca^2+^ and Na^+^ imaging discriminate between different Ca^2+^ permeation due to changes in [Na^+^]_o_. **A**, left, representative Ca^2+^ transients recorded in Hek293T cells expressing human NMDAR, elicited by coapplication of NMDA and glycine, in 0 mM [Ca^2+^]_o_ (grey), 1 mM [Ca^2+^]_o_ (blue) and 2 mM [Ca^2+^]_o_ (green). **A**, right, representative Na^+^ transients recorded in Hek293T cells expressing human NMDAR, same cells as A. **B** F_Ca_/F_Na_ for NMDAR in the three conditions of [Ca^2+^]_o_ (0, 1 and 2 mM, *n* = 8, 15 and 14, respectively; *p < 0.001). **C** linear relationships of the distributions of F_Ca_ and F_Na_ values for NMDAR in the three conditions of [Ca^2+^]_o_

**Fig. 3:**
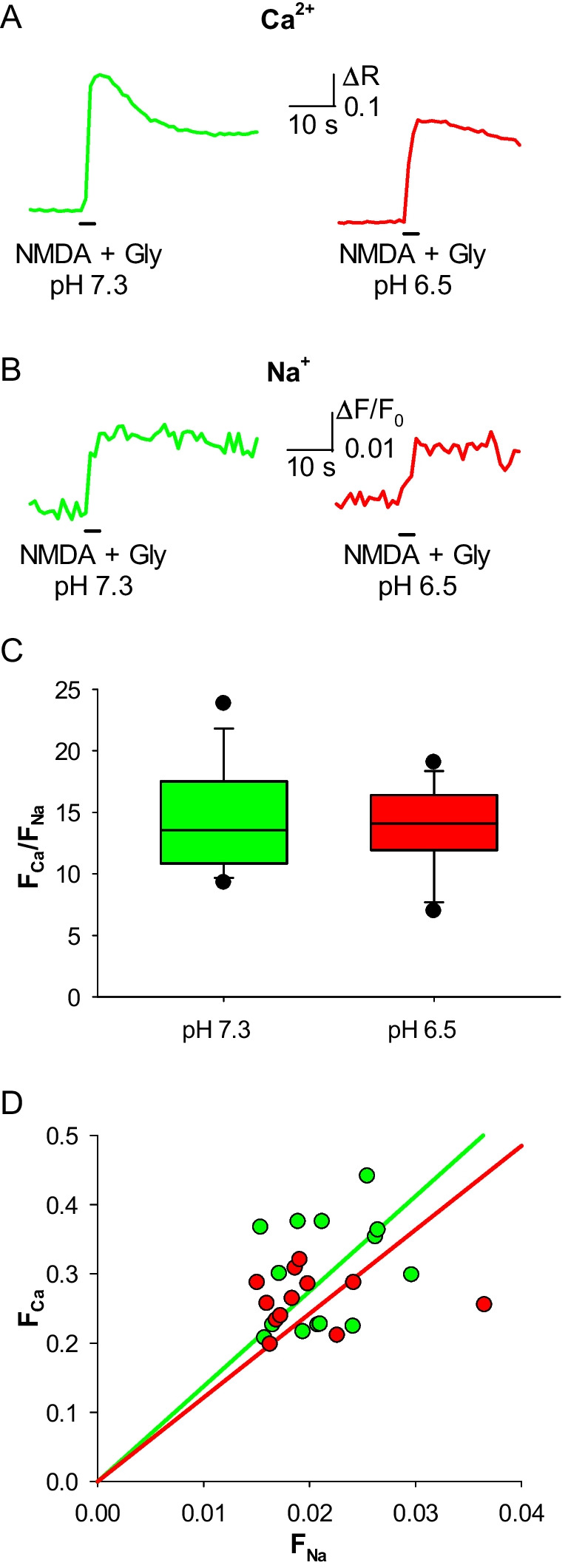
Simultaneous Ca^2+^ and Na^+^ imaging is not able to detect the effect of extracellular mild acidosis on NMDAR Ca^2+^ permeability. **A** representative Ca^2+^ transients recorded in Hek293T cells expressing human NMDAR, elicited by coapplication of NMDA and glycine in control cells (green, pH 7.4) and cells in a 6.5 pH extracellular solution (red). **B** representative Na^+^ transients recorded in Hek293T cells expressing human NMDAR, same cells as A. **C** F_Ca_/F_Na_ for NMDAR in control cells (green; *n* = 14) and in cells exposed to a 6.5 pH extracellular solution (red; *n* = 12); no significant difference. **D** linear relationships of the distributions of F_Ca_ and F_Na_ values for NMDAR in control cells (green) and in cells exposed to a 6.5 pH extracellular solution (red). Same cells as C

**Fig. 4:**
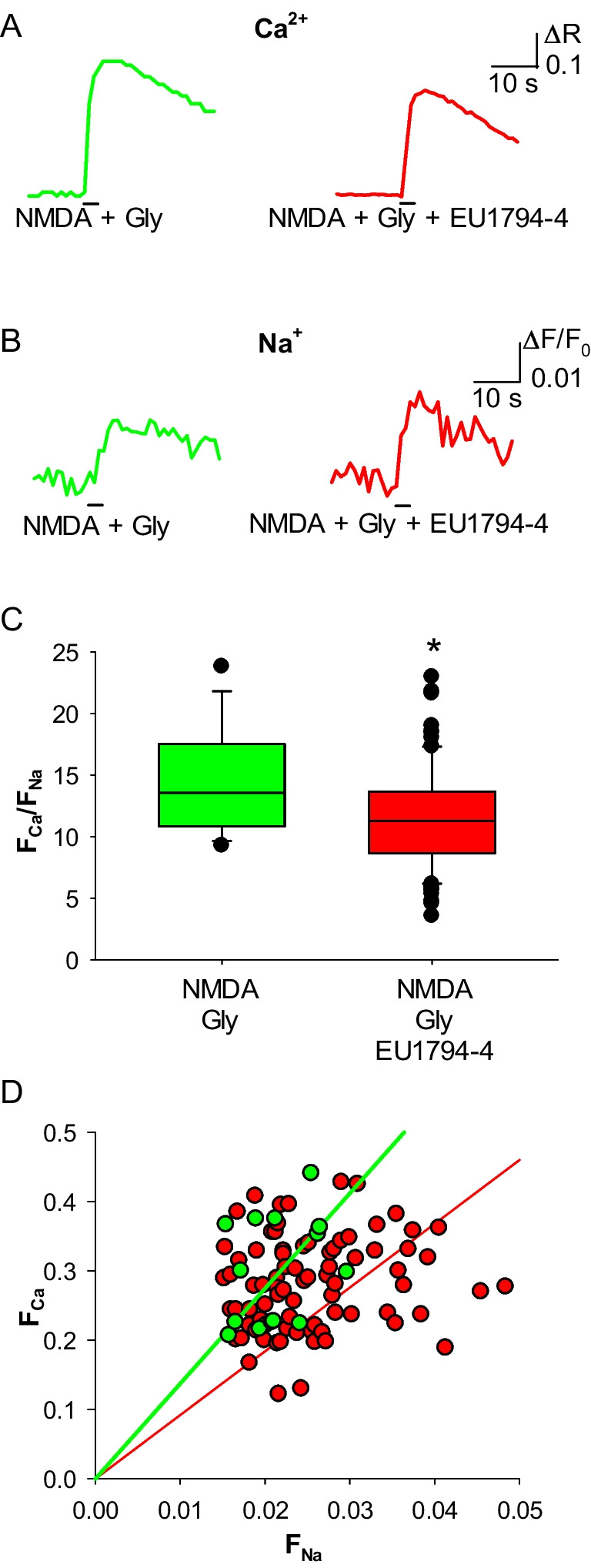
EU1794-4 reduces the Ca^2+^ permeability of human NMDAR. **A** representative Ca^2+^ transients recorded in Hek293T cells expressing human NMDAR, elicited by coapplication of NMDA and glycine in control cells (green) and in cells treated with EU1794-4 (30 μM, red). **B** representative Na^+^ transients recorded in Hek293T cells expressing human NMDAR, same cells as A. **C** F_Ca_/F_Na_ for NMDAR in control cells (green; *n* = 14) and in cells treated with EU1794-4 (red; *n* = 81). **D** linear relationships of the distributions of F_Ca_ and F_Na_ values for NMDAR in control cells (green) and in cells treated with EU1794-4 (red). Same cells as C

Ion imaging measures changes in the free intracellular concentration of a given ion. To extend its use to the quantification of ion fluxes through channels, it is crucial to demonstrate that this technique is able to highlight differences in the known Ca^2+^ permeabilities of distinct channels. Thus, we compared the human NMDAR with the human α_3_β_4_ nicotinic acetylcholine receptor (nAChR), a channel that has a lower P_f_ (2.7%[18]) and is well expressed in heterologous systems. We recorded, using Fura-2 and ING-2 as fluorophores, respectively the Ca^2+^ and Na^+^ influxes through human NMDARs and α_3_β_4_ nAChRs expressed in Hek293T cells (Fig. 1A, B). The NMDARs exhibited a higher F_Ca_/F_Na_ than α_3_β_4_ nAChRs (mean ratio: 14.5 ± 1.1 vs 5.8 ± 0.9; Fig. 1C, D), indicating the ability of this technique to discriminate between ion channels with different Ca^2+^ permeability. The same approach was able to evidentiate changes in Ca^2+^ permeation in the human NMDAR due to different [Ca^2+^]_o_ (0, 1, 2 mM Ca^2+^; Fig. 2A). F_Ca_/F_Na_ was 0 in the Ca^2+^-free condition (no Ca^2+^ signal was observed), has an intermediate value in the 1 mM Ca^2+^ condition and is higher in the physiological condition (mean ratio: 0 ± 0 vs 11.4 ± 0.7 vs 14.5 ± 1.1; Fig. 2B, C). Last, the effect of extracellular mild acidosis on the human NMDAR Ca^2+^ permeability was tested, given its ability to decrease the P_f_ of human NMDAR [11]. In this case, when pH_e_ was shifted from 7.3 to 6.5 during NMDA and glycine co-application on NMDAR-expressing Hek293T cells (Fig. 3A, B), there was no significant difference in F_Ca_/F_Na_ compared to control cells (mean ratio: 14.5 ± 1.1 vs 13.8 ± 1.0; Fig. 4 C, D), likely due to the strong influence of membrane potential on proton-channel interactions.

### EU1794-4 Decreases the Human NMDAR Ca^2+^ Permeability

**Fig. 5:**
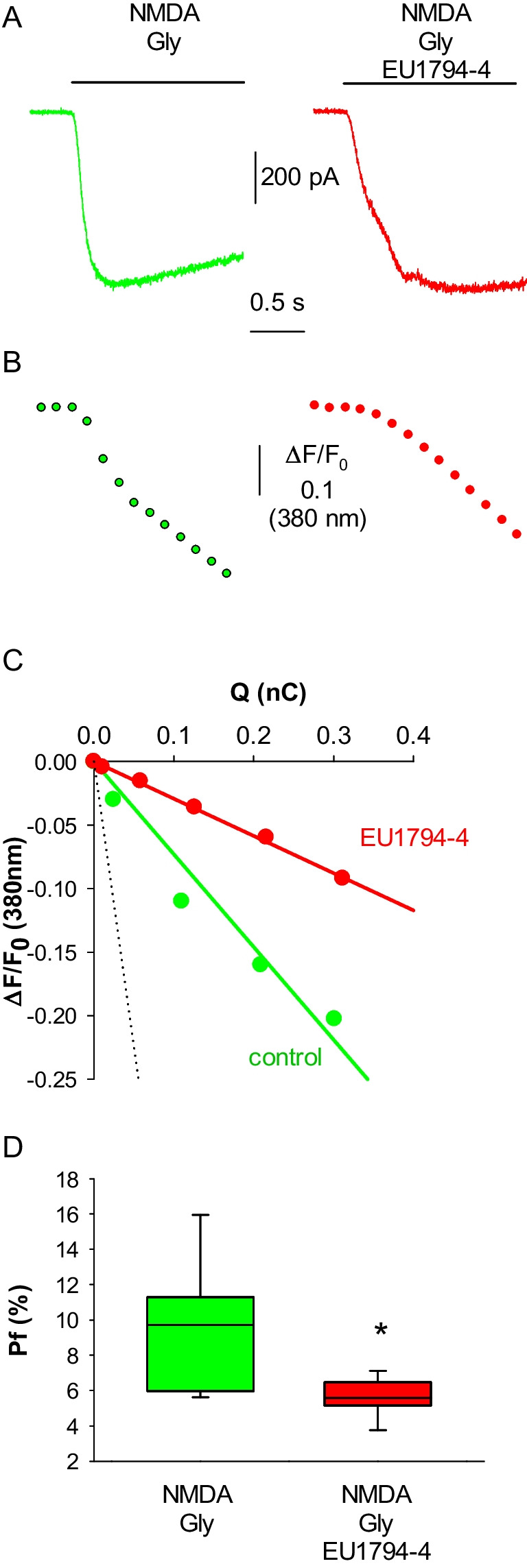
EU1794-4 reduces the fractional Ca^2+^ current (Pf) of the human NMDAR. **A** representative traces of the whole-cell currents elicited by the coapplication of NMDA and glycine in control cell (green) and in cell treated with EU1794-4 (30 μM, red). **B** representative traces of the Ca^2+^ transients recorded simultaneously with the current traces shown in **A**. At an excitation wavelength of 380 nm, [Ca^2+^]_i_ increase corresponds to a downward deflection of Fura-2 fluorescence emission. Traces in A and B are aligned and share the same temporal scale. **C** linear relationships between ΔF/F (data shown in panel B) and Q (time integral of the whole-cell current shown in panel A, calculated at the same times of ΔF/F values) obtained from the same cells as in A, B. The dashed line represents the mean slope obtained from calibration experiments (5.54 nC.^−1^; *n* = 9). D, box plot of P_f_ values in control cells (green; *n* = 8) and in cells treated with EU1794-4 (red; n = 8; *p = 0.025)

The negative allosteric modulator EU1794-4 has recently been shown to reduce the single-channel conductance and Ca^2+^ permeability of NMDAR, assessed by analyzing the shift of the reversal potential [13]. In its presence (30 μM, Fig. 4A, B) we recorded a significant reduction of the F_Ca_/F_Na_ in Hek293T cells expressing the NMDAR, compared to untreated cells (mean ratio: 14.5 ± 1.1 vs 11.6 ± 0.5; Fig. 4C, D). To further detail the effect of EU1794-4 on the Ca^2+^ permeability of human NMDAR we measured, for the first time, its P_f_, simultaneously recording NMDA-evoked whole-cell currents and variations of intracellular free Ca^2+^ concentration ([Ca^2+^]_i_, Fig. 5A, B): the mean P_f_ value of NMDAR in control cells was 9.4 ± 1.2%, while the P_f_ value of NMDAR in cells treated for 3 s with 30 μM EU1794-4 was significantly reduced to 5.7 ± 0.4% (Fig. 5C, D). The current density values were measured in distinct transfected cells and did not show any significant difference (mean values 34 ± 4 pA/pF *n* = 10 and 40 ± 3 pA/pF, *n* = 10, in the absence or presence of EU1794-4, respectively).

### EU1794-4 Reduces the Ca^2+^ Entry Through NMDAR in Human iPSCs-derived Neurons

**Fig. 6:**
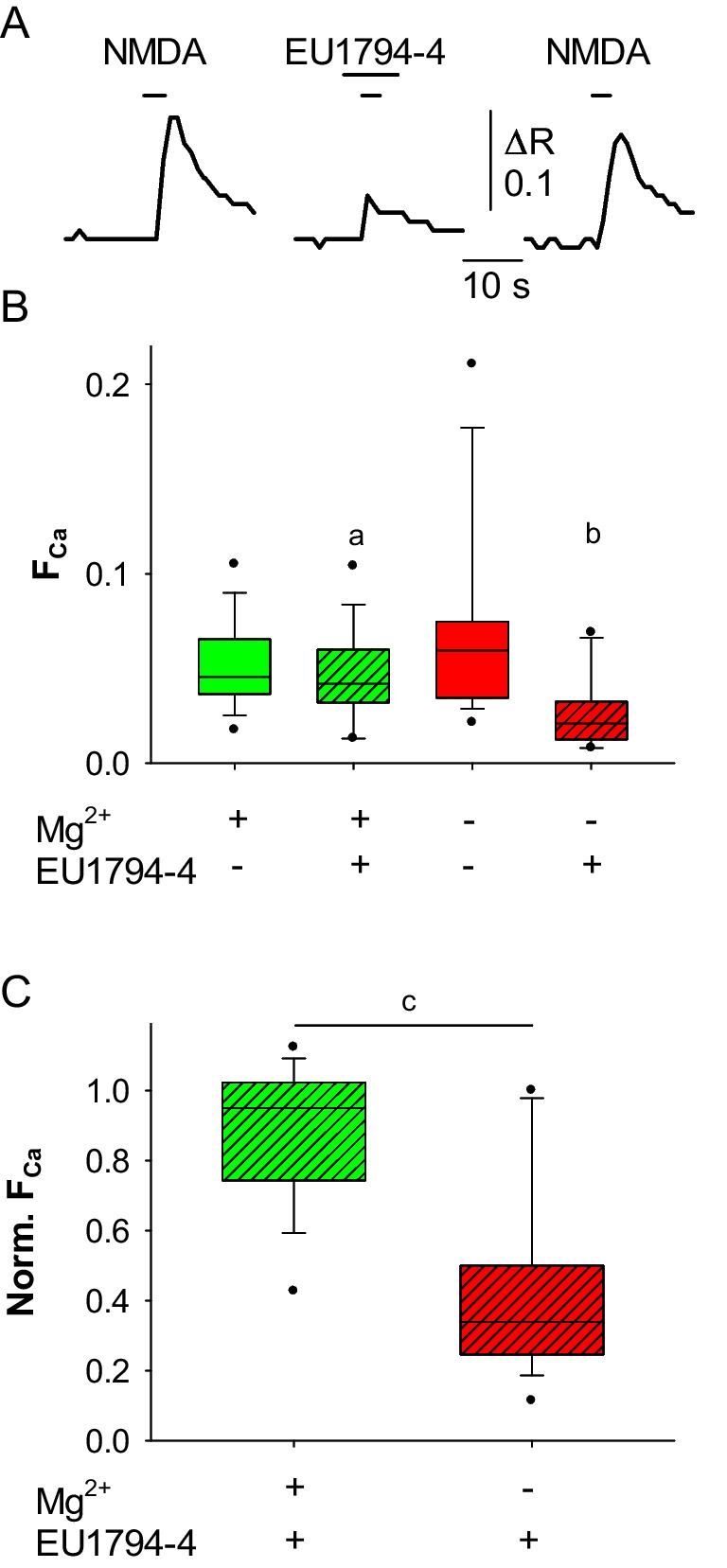
EU1794-4 reduces NMDAR-mediated Ca^2+^ influx in human neurons derived from iPSCs, in the presence or absence of Mg^2+^. **A** representative Ca^2+^ transients elicited by coapplication of NMDA and glycine in human neurons before (left), during (center) and after (left) treatment with EU1794-4 (in Mg^2+^-free solution). **B** box plot of the NMDA-induced F_Ca_ values in the presence (green; *n* = 15) or absence of 2 mM extracellular Mg^2+^ (red; *n* = 16) in control cells (empty boxes) and in cells treated with EU1794-4 (hatched boxes; a: *p* = 0.011, b: *p* < 0.001). **C** box plot of the normalized NMDA-induced F_Ca_ values in the presence (green; *n* = 15) or in the absence (red; *n* = 16) of 2 mM extracellular Mg^2+^ in neurons treated with EU1794-4 (c: *p* < 0.001)

To study the effect of EU1794-4 on human NMDAR in a more physiological context, we measured the Ca^2+^ and Na^+^ signals elicited by NMDAR activation in human neurons derived from iPSCs (Supplementary Fig. and Fig. 6A). In these cells Na^+^ transients were not detectable, likely due to the small amplitude of NMDA-mediated currents and the consequent subthreshold increase of [Na^+^]_i_. By contrast, we recorded a significant reduction of the F_Ca_ in neurons treated with the EU1794-4 compared to control cells (mean: 0.051 ± 0.005 vs 0.046 ± 0.006; Fig. 6B, green boxes). This difference was more evident in the absence of extracellular Mg^2+^ (mean: 0.07 ± 0.01 vs 0.027 ± 0.005; Fig. 6B, red boxes). The normalized mean amplitude of Ca^2+^ transients was very different in the presence or absence of Mg^2+^ (mean: 0.87 ± 0.05 vs 0.42 ± 0.07; Fig. 6C), suggesting a competition mechanism between this ion and the positively charged EU1794-4.

## Discussion

Excessive Ca^2+^ influx through NMDARs has been linked to neuronal damage in several pathologies [19–21], and efforts have been made to lower the Ca^2+^-dependent excitotoxicity [22–24], providing clinically approved drugs targeted to NMDARs, such as memantine [25, 26]. However, the blocking of these receptor channels is not a suitable therapeutic strategy, given the high and relevant adverse effects [27, 28]. In this study we demonstrate the possibility of pharmacologically lowering NMDAR-mediated Ca^2+^ entry in human iPSC-derived neurons by modulating the ion selectivity of human NMDARs, altering their Ca^2+^/Na^+^ influx ratio without the need to block these essential receptor-channels. Our analysis is based on a new experimental approach, recording simultaneously the intracellular free Ca^2+^ and Na^+^ concentrations ([Ca^2+^]_i_ and [Na^+^]_i_, respectively. The two classical methods to study Ca^2+^ permeability of a cationic channel are the measure of the shift of the reversal potential upon changes of the [Ca^2+^]_o_ [29], and the measure of the fractional Ca^2+^ current (P_f_, i.e. the percentage of the total current carried by Ca^2+^ ions) by the simultaneous recording of the transmembrane current and of the changes in [Ca^2+^]_i_ [7]. This second method, not relying on any theoretical assumption [30], is highly reliable, and it has been used to measure the P_f_ values for a large number of ligand-gated ion channels [9, 18, 31]. However, it is also extremely time-consuming, and thus not suitable for screening the effects of multiple candidate drugs. Our new approach is no longer centered on directly measuring ionic fluxes, but on the changes of [Ca^2+^]_i_ and [Na^+^]_i_ due to fluxes, measured simultaneously by fluoresce microscopy. This technique, already described in different contexts [14], was used here for the first time to evaluate the Ca^2+^ permeability of ligand-gated ion channels. Of course, using changes in intracellular ion concentrations to compare ionic fluxes is not an obvious operation, given the possibility that other mechanisms could modulate concentrations, such as ion buffering and active transport. Thus, before applying this approach to test whether a drug is able to affect the ion selectivity of a channel, it must be quantitatively validated. Thus, we showed that this method was able to discriminate between different ion channels already known to exhibit different P_f_ values, such as human α_3_β_4_ nAChRs (P_f_ = 2.7%; [18, 32]) and human NR1/NR2A NMDARs (P_f_ = 11%; [9]), and that it was possible to clearly separate different Ca^2+^ entries through human NMDARs due to changes in [Ca^2+^]_o_. Interestingly, doubling the [Ca^2+^]_o_ (from 1 to 2 mM) produced a 27% increase in F_Ca_/F_Na_ value. This non-linear relationship may in principle be due to different mechanisms, even acting simultaneously, such as ion selectivity properties of NMDAR and differential kinetics of Ca^2+^ buffering and/or pumping at different [Ca^2+^]_o_. Anyway, channel activation is much faster than the mechanisms underlying ion buffering and removal, and consequently at least the earliest phases of the concentration transient are determined by ion entry. Furthermore, the comparison between ion concentrations is made in a well-defined cellular context in which the buffering and removal mechanisms are maintained. Thus, our data, taken together, demonstrate that it is possible to monitor [Ca^2+^]_i_ and [Na^+^]_i_ simultaneously, without fluorescence signal overlapping, with a suitable time resolution, allowing the detection of the differential modulation of the influx of these two ions through the same channel. Of course, the signal-to-noise ratio is much higher for Ca^2+^ than for Na^+^ signals, as evidenced by the experimental traces and expected due to the much higher intracellular concentration of Na^+^, which is scarcely affected by the inward Na^+^ currents.

By contrast, our experimental approach was not able to replicate our previous findings about the ability of mild acidosis (pH 6.5; [11]) to reduce the P_f_ of human NMDARs. We speculate that this discrepancy might be due to the fact that the present measurements were made on non voltage-clamped cells, and that the interaction of protons with the NMDAR channel pore could be sensitive to membrane potential, even though the inhibitory effect of pH on NMDA kinetics is voltage independent [33]. The reduction of P_f_ due to acidosis was recorded on cells clamped at -70 mV [11], while Hek293 T cells have a much more depolarized resting potential, between -20 and -30 mV. This observation indicates that the modulation of the biophysical properties of ion channels must be analyzed considering all the surrounding contexts and parameters. The same concept arises from our study of the effect of a recently developed compound, an NMDAR-selective negative allosteric modulator, EU1794-4, able to reduce the Ca^2+^ permeability of NMDAR [13]. In our hands this molecule was able to reduce the F_Ca_/F_Na_ ratio of human NMDARs, confirming previous results [13]. For the first time, we measured the P_f_ of human NMDAR in the presence of EU1794-4, revealing a relevant 40% reduction in comparison to control conditions, similar to the reduction of the permeability ratio P_Ca_/P_Na_ observed by the Traynelis group [12]. With the same drug, we were able to reduce NMDA-dependent Ca^2+^ entry in human iPSC-derived neurons. In these cells, it was not possible to reliably measure changes in the F_Ca_/F_Na_ ratio, due to undetectable [Na^+^]_i_ variations. This technical limit is probably linked to high values of [Na^+^]_i_ in our iPSC-derived neurons, and/or to the small NMDA-mediated current amplitudes in these cells. The inhibitory effect of EU1794-4 on NMDA-mediated Ca^2+^ entry depends on extracellular Mg^2+^, being much stronger in its absence (58% inhibition vs 13%), suggesting a possible interaction between Mg^2+^ and EU1794-4. This observation about a different functional behavior of an allosteric modulator in different ionic conditions must be kept in mind, given the high number of studies on NMDARs done in the absence of extracellular Mg^2+^. Even if the EU1794-4-mediated inhibition of Ca^2+^ entry in neurons was smaller in the presence of Mg^2+^, this result is still very interesting: to our knowledge this compound is the first to be shown to reduce NMDA-mediated Ca^2+^ influx in human iPSC-derived neurons without blocking these receptor-channels, opening a new field of possible therapeutic tools to fight Ca^2+^- and NMDA-dependent excitotoxicity, and thus neurodegeneration, in a wide number of neuropathologies.

### Supplementary Information

Below is the link to the electronic supplementary material.

Supplementary file1 (DOCX 1079 kb)

## Data Availability

The datasets generated during and/or analyzed during the current study are available as DOI: 10.12751/g-node.z6m3kt.

## Abbreviations

ACh: Acetylcholine
ALS: Amyotrophic lateral sclerosis
CNS: Central nervous system
DMEM: Dulbecco’s modified eagle medium
FBS: Fetal bovine serum
HEPES: N’-2-Hydroxyethylpiperazine-N’-2 ethanesulphonic acid
ING-2: Ion Natrium Green-2
iPSC: Induced pluripotent stem cell
nAChRs: Nicotinic acetylcholine receptors
NES: Normal external solution
NMDA: N-methyl-D-aspartate
NMDAR: N-methyl-D-aspartate receptor
P_f_: Fractional Ca^2+^ current
ROS: Reactive oxygen species
