# Supplementary material for: Selective Reduction of Ca2+ Entry Through the Human NMDA Receptor: a Quantitative Study by Simultaneous Ca2+ and Na+ Imaging
Source: Mol Neurobiol. 2024 Jan 19;61(8):5841–50. doi: 10.1007/s12035-024-03944-9 (PMC11249768; doi:10.1007/s12035-024-03944-9)
Supplement: Supplementary file 1 — Supplementary file1 (DOCX 1079 kb) [file 12035_2024_3944_MOESM1_ESM.docx]

**Selective reduction of Ca^2+^ entry through the human NMDA receptor:**

**a quantitative study by simultaneous Ca^2+^ and Na^+^ imaging**

Tiziano D’Andrea^1^, Maria Cristina Benedetti^2,3^, Lucia Monaco^1^, Alessandro Rosa^2,3^, Sergio Fucile^1,4^

^1^Department of Physiology and Pharmacology, Sapienza University of Rome, Rome, Italy.

^2^Department of Biology and Biotechnologies “Charles Darwin”, Sapienza University of Rome, Rome, Italy.

^3^Center for Life Nano- & Neuro-Science, Fondazione Istituto Italiano di Tecnologia (IIT), Rome, Italy.

^4^IRCCS Neuromed, Pozzilli (Is), Italy

**Corresponding Author**

Prof. Sergio Fucile; email: [sergio.fucile@uniroma1.it](mailto:sergio.fucile@uniroma1.it)

SUPPLEMENTARY FIGURE





Supplementary figure: representative traces of the functional activity recorded from human iPSC-derived spinal motoneurons after a differentiation period of 21 days.

A, action potentials evoked by current injection (90 pA). B, whole-cell current evoked by glycine administration (30 μM, 3 s, -70 mV holding potential). C, whole-cell current evoked by glutamate administration (1 mM, 3 s, -70 mV holding potential). D, whole-cell current evoked by GABA administration (100 μM, 3 s, -70 mV holding potential). Data representative of 6 human iPSC-derived neurons (mean capacitance: 38 ± 6 pF; mean resting potential: -59 ± 2 mV; mean action potential threshold: -33 ± 2 mV).
